# Author Correction: Molecular basis for bacterial *N*-glycosylation by a soluble HMW1C-like *N*-glycosyltransferase

**DOI:** 10.1038/s41467-024-45708-y

**Published:** 2024-02-13

**Authors:** Beatriz Piniello, Javier Macías-León, Shun Miyazaki, Ana García-García, Ismael Compañón, Mattia Ghirardello, Víctor Taleb, Billy Veloz, Francisco Corzana, Atsushi Miyagawa, Carme Rovira, Ramon Hurtado-Guerrero

**Affiliations:** 1 Departament de Química Inorgànica i Orgànica (Secció de Química Orgànica) and Institut de Química Teòrica i Computacional (IQTCUB), Universitat de Barcelona, Martí i Franquès 1, 08028 Barcelona, Spain; 2 Institute of Biocomputation and Physics of Complex Systems, University of Zaragoza, Mariano Esquillor s/n, Campus Rio Ebro, Edificio I+D, Zaragoza, Spain; 3 Department of Life Science and Applied Chemistry, Nagoya Institute of Technology, Gokiso-cho, Showa-ku, Nagoya, 466-8555 Japan; 4 Departamento de Química, Universidad de La Rioja, Centro de Investigación en Síntesis Química, E−26006 Logroño, Spain; 5 Institució Catalana de Recerca i Estudis Avançats (ICREA), Passeig Lluís Companys 23, 08010 Barcelona, Spain; 6 Copenhagen Center for Glycomics, Department of Cellular and Molecular Medicine, University of Copenhagen, Copenhagen, Denmark; 7 Fundación ARAID, 50018 Zaragoza, Spain

**Keywords:** X-ray crystallography, Computational biology and bioinformatics, Enzymes

Correction to: *Nature Communications* 10.1038/s41467-023-41238-1, published online 18 September 2023

The original version of this Article contained an error in the Acknowledgements, which incorrectly omitted from the end the following: “The authors would like to thank the technical support provided by the Barcelona Supercomputing Center (BSC) and Red Nacional de Supercomputación (RES; application BCV-2023-2-0016) for computer resources at MareNostrum IV and CTE-Power supercomputers.”. This has been corrected in both the PDF and HTML versions of the Article.

The original version of this Article contained an error in the Methods which incorrectly read “Docking of UDP-Glc into the structure of quitar subrallado AaNGT”. The correct version replaces this sentence with “Docking of UDP-Glc into the structure of AaNGT”. This has been corrected in both the PDF and HTML versions of the Article.

