# Supplementary material for: Molecular basis for bacterial N-glycosylation by a soluble HMW1C-like N-glycosyltransferase
Source: Nat Commun. 2023 Sep 18;14:5785. doi: 10.1038/s41467-023-41238-1 (PMC10507012; doi:10.1038/s41467-023-41238-1)
Supplement: Supplementary file 1 — Supplementary Information [file 41467_2023_41238_MOESM1_ESM.pdf]

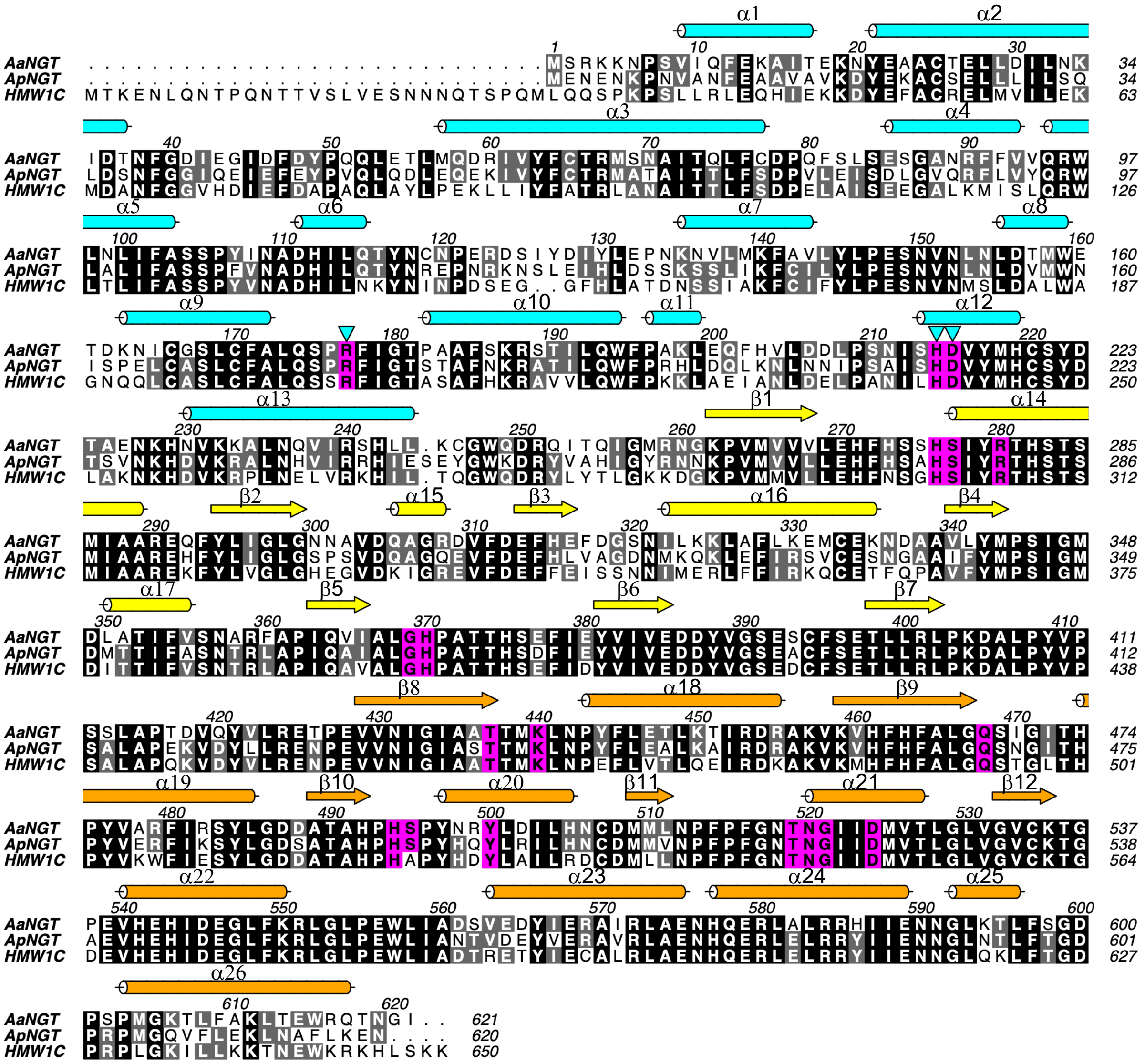

Supplementary Figure 1. Multiple sequence alignment between *Aa*NGT, *Ap*NGT and HMW1C. Residues are color-coded by degree of sequence conservation where black, grey and white colours denote high similarity, medium similarity and dissimilarity, respectively. The secondary structure elements (α-helices and β-strands) based on the *Aa*NGT domains are shown above the sequence. The N-terminal AAD, the N-terminal Rossmann and C-terminal Rossmann fold subdomains are colored in cyan, yellow and orange, respectively. The residues involved in recognition of the ligands are highlighted in magenta. Residues mutated in this work are indicated with an inverted cyan triangle. Note that most of the residues are conserved between the NGTs.

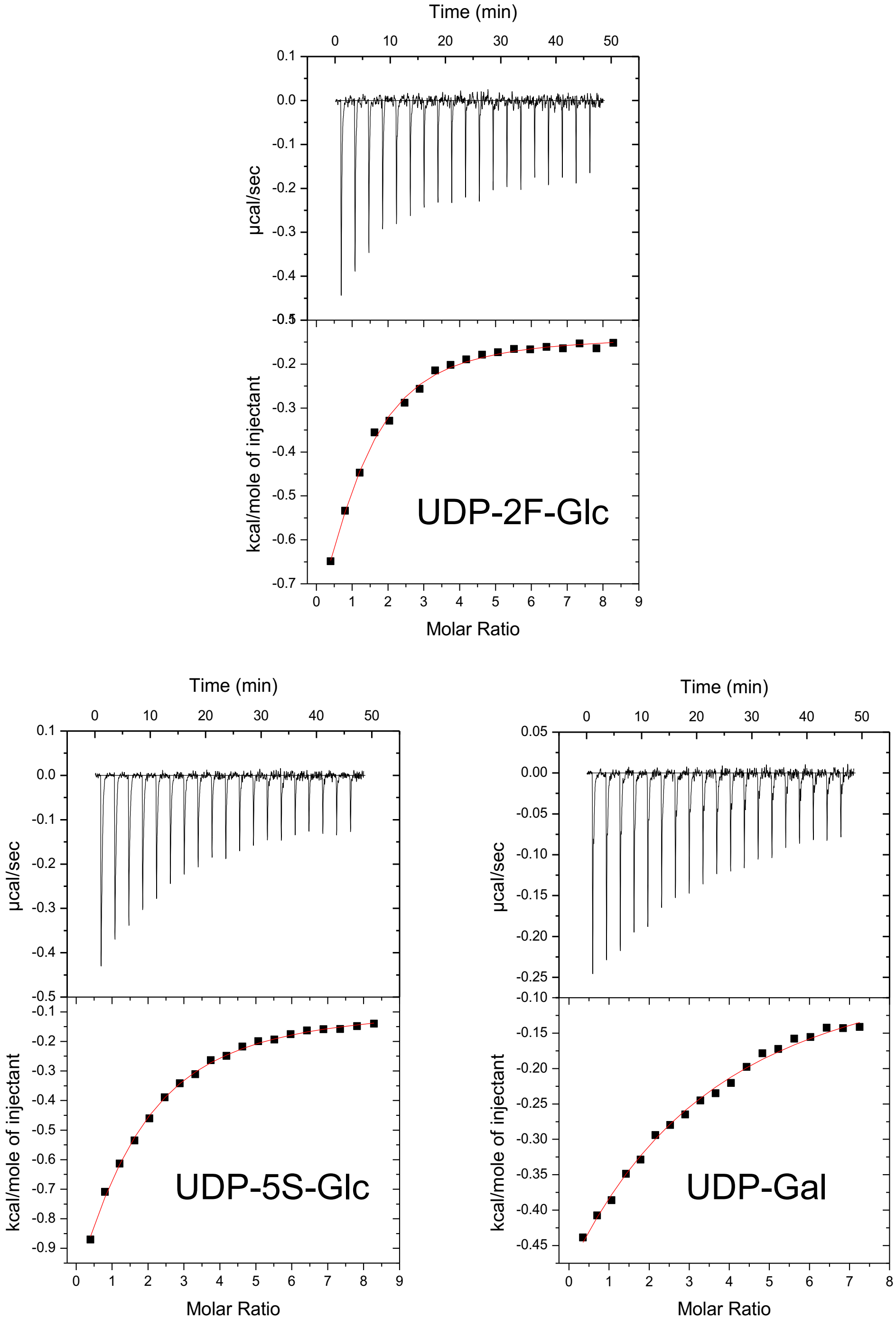

Supplementary Figure 2. ITC data for the binding of UDP-Gal, UDP-2F-Glc and UDP-5S-Glc to *Aa*NGT. Top: raw thermogram (thermal power versus time). Bottom: binding isotherm (normalized heats versus molar ratio). See Supplementary Table 2 for the thermodynamic and K_D values for all the experiments. The experiments were repeated at least 2 times independently with similar results, and one representative plot for each experiment is shown. Source data are provided as a Source Data file.

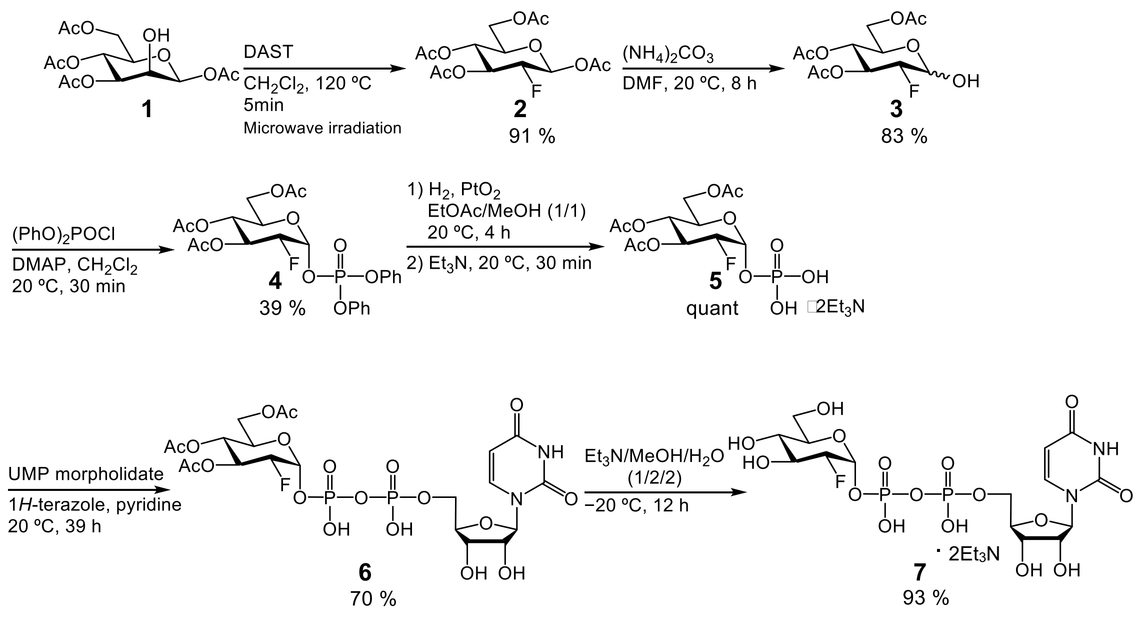

Supplementary Figure 3. Synthetic route towards UDP-2F-Glc.

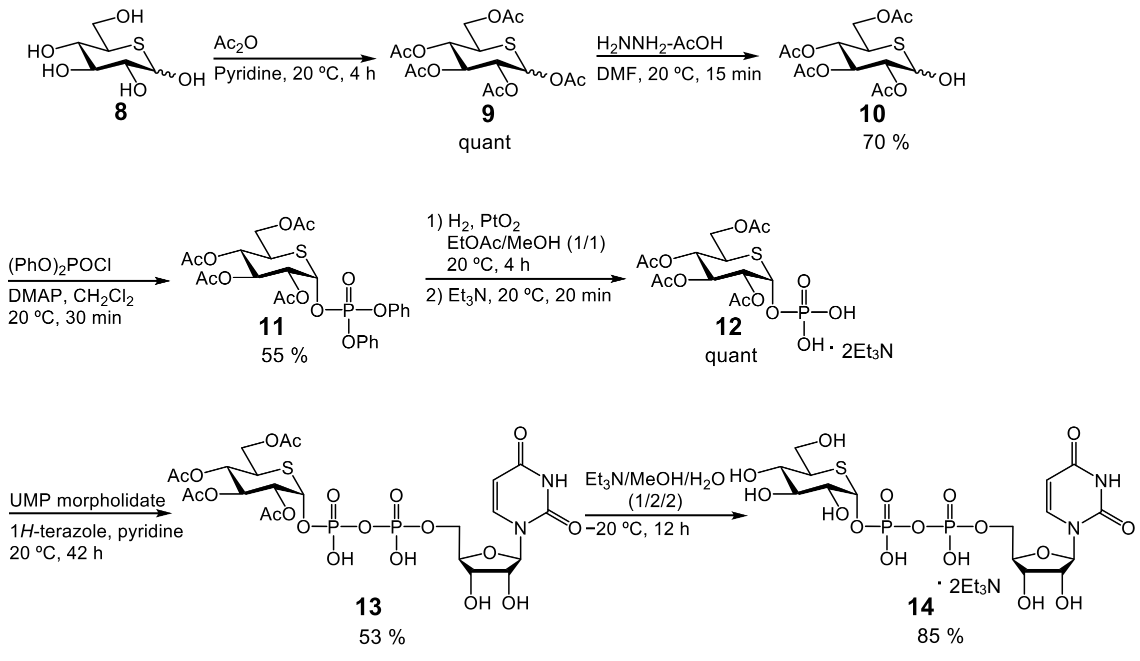

Supplementary Figure 4. Synthetic route towards UDP-5S-Glc.

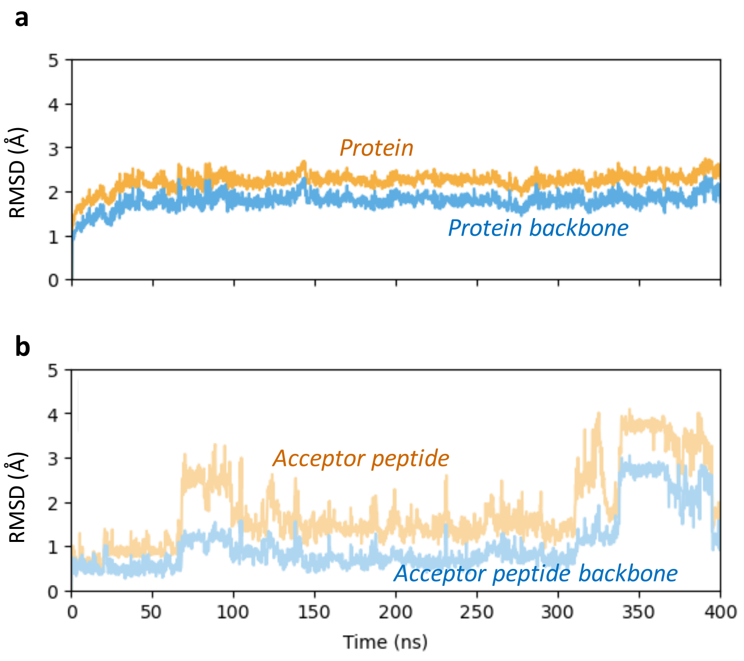

Supplementary Figure 5. RMSD along the classical MD simulation of the Michaelis complex. **a** Protein. **b** Acceptor peptide. Hydrogen atoms are not included. Graphics obtained with Ambertools. Plot data provided in Source Data file.

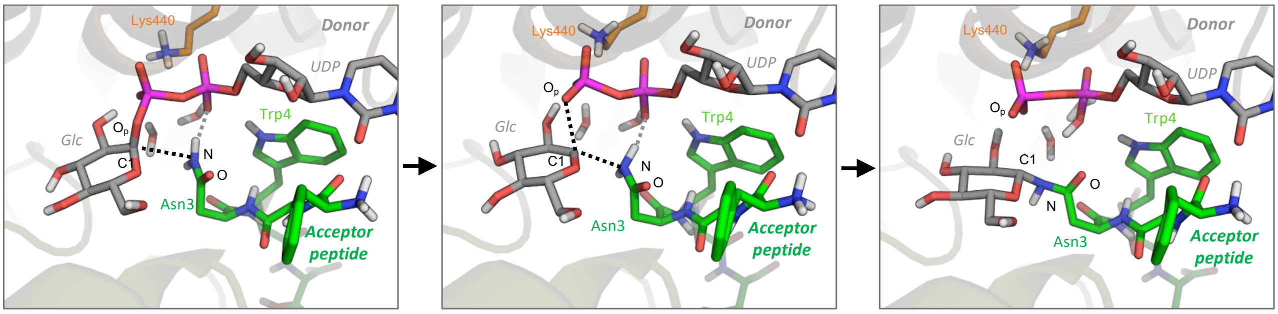

Supplementary Figure 6. Reaction pathway starting from the acceptor asparagine (Asn3^{P}) in the common amide form. Representative structures along the reaction pathway obtained from QM/MM metadynamics simulations.

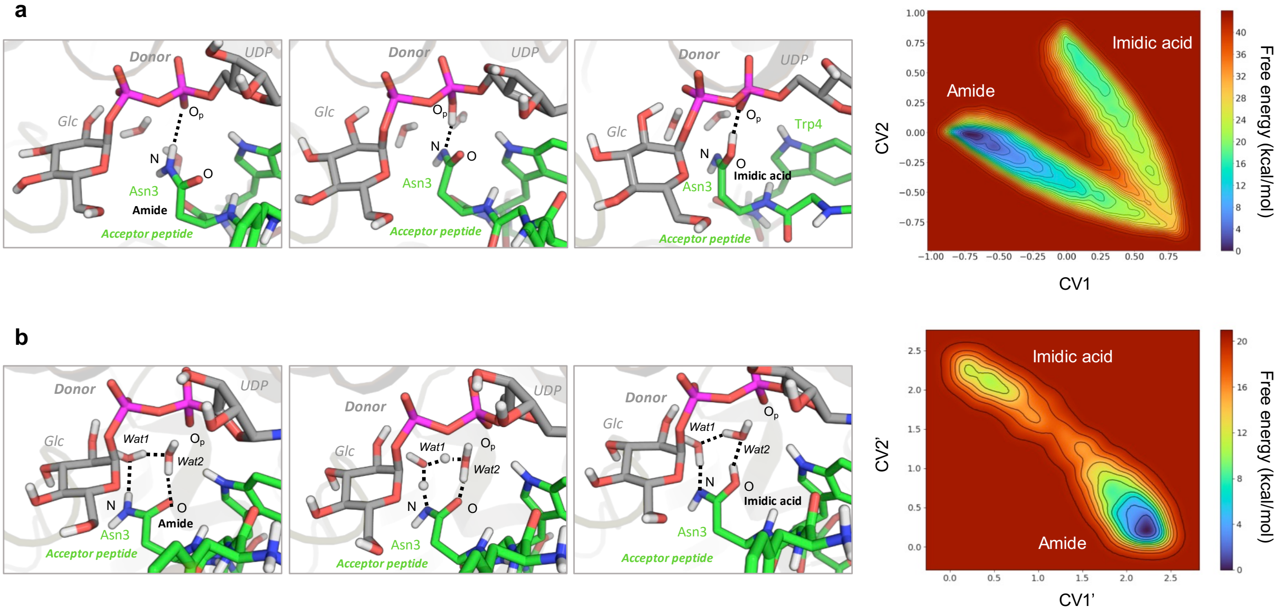

Supplementary Figure 7. Tautomerization of the acceptor peptide asparagine (Asn3^{P}) in the active site of *Aa*NGT. Representative structures along the tautomerization process, obtained from QM/MM metadynamics simulations. **a** Tautomerization mediated by the α-phosphate. **b** Tautomerization mediated by active site water molecules. FES data have been deposited in Zenodo (https://doi.org/10.5281/zenodo.8081487).

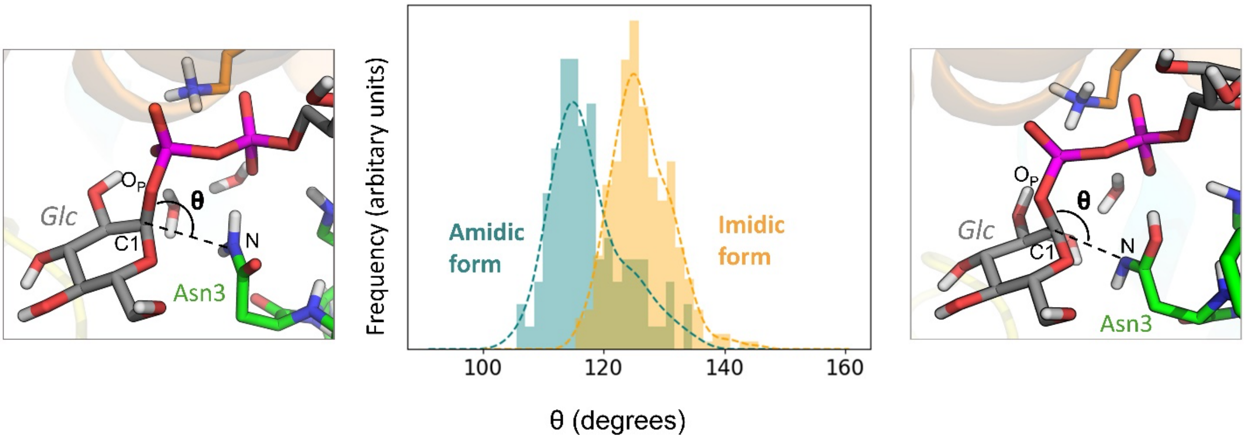

Supplementary Figure 8. Frequency distribution of the N-C1-O_P angle in the Michaelis complex of *Aa*NGT. Both amidic and imidic forms of Asn3 are compared. Plot data in Source Data file.

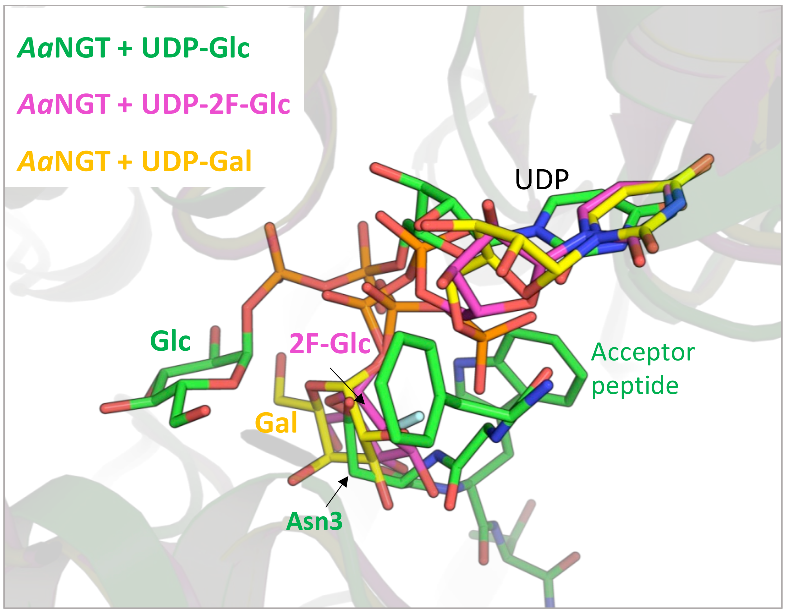

Supplementary Figure 9. Overlay of structures of *Aa*NGT in complex with UDP-Glc, UDP-Gal and UDP-2F-Glc. The structures with UDP-Gal and UDP-2F-Glc were obtained by X-ray crystallography. The structure with UDP-Glc was modeled by molecular docking, followed by molecular dynamics, using the structures obtained in this work.

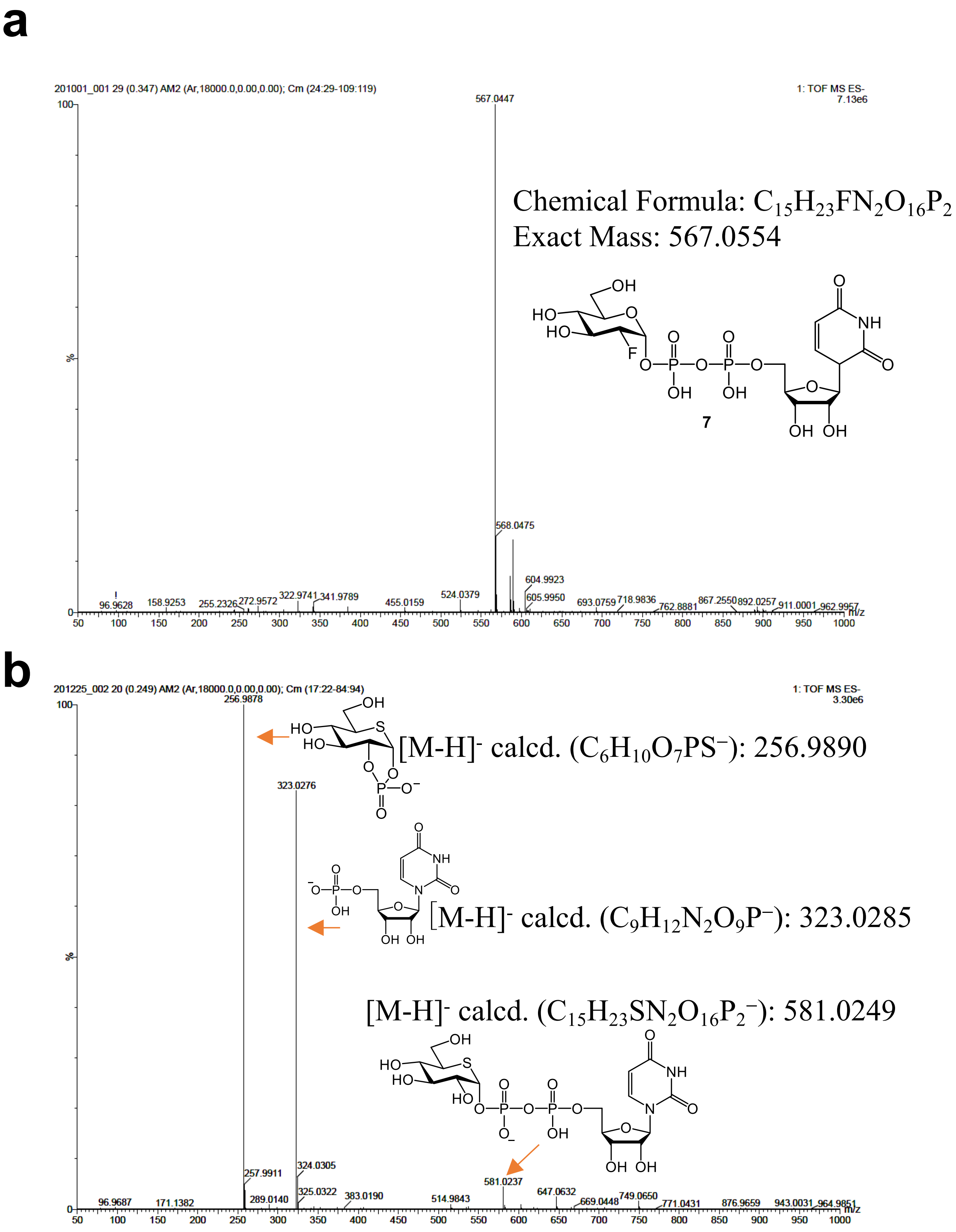

Supplementary Figure 10. ESI-high resolution mass spectrometry (ESI-HRMS) spectra. a ESI-HRMS spectrum of UDP-2F-Glc and **b** ESR-HRMS spectrum of UDP-5S-Glc.

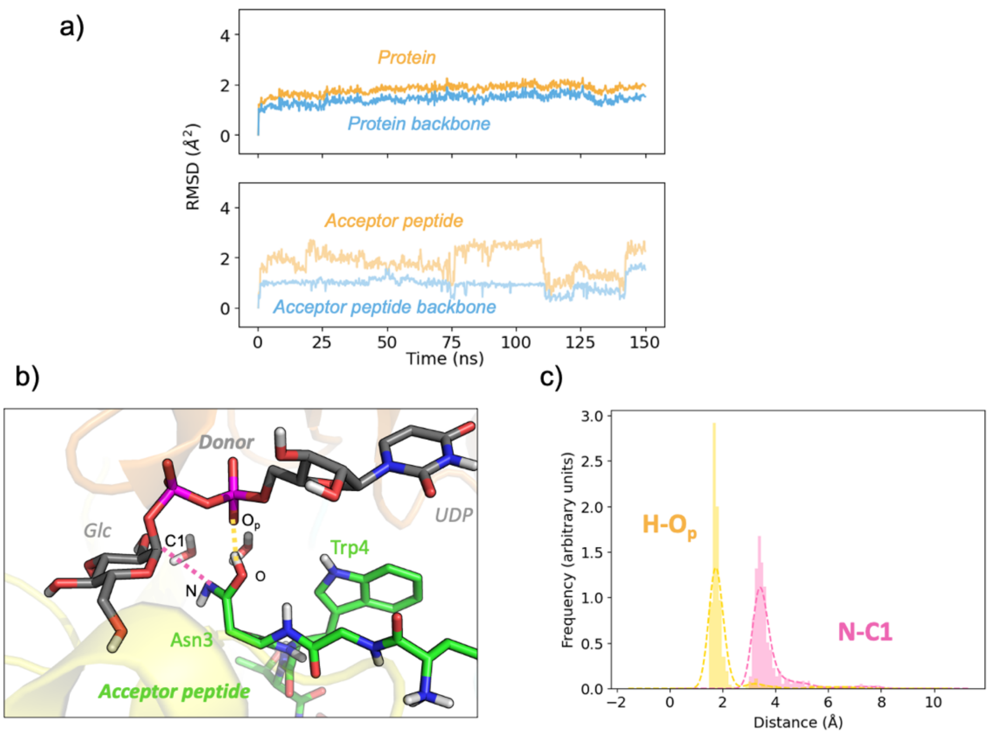

Supplementary Figure 11. Results of the classical MD simulation of AaNGT in complex with UDP-Glc and the acceptor peptide, considering Asn3^P in the imidic acid form. **a** RMSD evolution of the protein (top) and the acceptor peptide (bottom). Hydrogen atoms are not included. **b** Representative structure of the active site. Obtained with Ambertools. **c** Frequency distribution of the distance of nucleophilic attack (N-C1) and the hydrogen bond between the hydroxyl group of Asn3^P and the oxygen atom of the alpha phosphate (H-O$_p$). Plot data in Source Data file.

Supplementary Table 1. Kinetic parameters of UDP-Glu and the peptide FGNWTT with *Aa*NGT. Error values represent the standard error calculated by the GraphPad Prism fit. Source data are provided as a Source Data file.

Substrate	V_{max} (nmol · min^{-1}·mg^{-1})	K_m (µM)	k_{cat} (min^{-1})	k_{cat}/K_m (min^{-1}·µM^{-1})
**UDP-Glc**	278 ± 26	90 ± 30	20 ± 2	0.22
**FGNWTT (*Aa*NGT wild type)**	222 ± 8	79 ± 11	16 ± 1	0.20
**FGNWTT (H214A mutant)**	21 ± 7	202 ± 21	1.4 ± 0.1	0.007

Supplementary Table 2. Thermodynamic parameters for the binding of different ligands to *Aa*NGT. K_D is the dissociation constant (defined as 1/K), and ΔG, ΔH and -TΔS are the thermodynamic parameters. The stoichiometry of binding for most cases was found to be approximately ≈1:1. Error values represent the error calculated through iteration fit of the data sets by the Origin 7 (Microcal). Source data are provided as a Source Data file.

Ligand	K_D (µM)	ΔH (kcal/mol)	-T*ΔS (kcal/mol)	ΔG (kcal/mol)	n
**UDP**	35 ± 8.7	-5.08 ± 0.54	-0.99	-6.07	1.07
**UDP-2F-Glc**	103.7 ± 21.35	-1.17 ± 0.22	-4.26	-5.43	1
**UDP-5S-Glc**	203.5 ± 37.7	-2.48 ± 0.62	-2.55	-5.03	1.07
**UDP-Gal**	630 ± 91.85	-2.41 ± 0.35	-1.95	-4.36	1.39

Supplementary Table 3. Relevant distances of the main States along the reaction coordinate. Values (with standard deviation, in Angstrom) obtained from the computed reaction free energy landscape, averaging over frames within 1 kcal/mol of the corresponding energy minimum (MC and P). TS values were obtained from committor analysis. Structure coordinates and further data can be found in Zenodo https://doi.org/10.5281/zenodo.8081487).

Distance[a]	**MC**	**TS**	**P**
N-C1	3.09 ± 0.08	2.08	1.47 ± 0.04
C1-O_P	1.46 ± 0.04	2.16	3.31 ± 0.05
H···O_α	1.72 ± 0.15	1.12	1.01 ± 0.03
O-H	1.01 ± 0.03	1.34	1.71 ± 0.14

[a] The depicted connectivity refers to the MC state.
